# Functional annotation of lung cancer‒associated genetic variants by cell type‒specific epigenome and long-range chromatin interactome

**DOI:** 10.5808/gi.20073

**Published:** 2021-03-25

**Authors:** Andrew J. Lee, Inkyung Jung

**Affiliations:** Department of Biological Sciences, Korea Advanced Institute of Science and Technology (KAIST), Daejeon 34141, Korea

**Keywords:** 3D chromatin interaction, *cis*-regulatory element, genome-wide association study, lung cancer, single-cell RNA sequencing, single nucleotide polymorphism

## Abstract

Functional interpretation of noncoding genetic variants associated with complex human diseases and traits remains a challenge. In an effort to enhance our understanding of common germline variants associated with lung cancer, we categorize regulatory elements based on eight major cell types of human lung tissue. Our results show that 21.68% of lung cancer‒associated risk variants are linked to noncoding regulatory elements, nearly half of which are cell type‒specific. Integrative analysis of high-resolution long-range chromatin interactome maps and single-cell RNA-sequencing data of lung tumors uncovers number of putative target genes of these variants and functionally relevant cell types, which display a potential biological link to cancer susceptibility. The present study greatly expands the scope of functional annotation of lung cancer‒associated genetic risk factors and dictates probable cell types involved in lung carcinogenesis.

## Introduction

Gene regulation is a critical biological process that determines cellular identity and function. Systematic investigation of chromatin architecture has shown that the spatiotemporal gene regulation process is tightly controlled by *cis*-regulatory elements (*c*REs), which modulate the activities of spatially distant promoters [1-4]. Previous studies have reported that *c*REs are highly dynamic genomic entities whose dysregulation is associated with various human disorders, including genetic and complex diseases [5-7]. This is well described in the 2019 professional release of the human gene mutation database (HGMD), where more than 4,500 disease-associated mutations are in regulatory sequences [8]. The distal target genes of *c*REs harboring genetic mutations have been identified as causal elements in human diseases. The representative example is polydactyly syndrome, a congenital limb malformation that results from point mutations in a *Shh* regulatory element [9]. However, the identification of such long-range regulation is difficult since *c*REs may regulate genes located beyond large genomic distances. In light of this, Hi-C is a novel technique that enables the investigation of genome-wide, all-to-all chromatin interactions and has substantially improved our view of *c*REs on long-range gene expression control through 3D chromatin organization [10-14].

The pathogenesis of complex diseases is an outcome of a heterogeneous cell population and various causal genetic variants. The causal genetic variants and the functional cell type in which these disease-associated variants may be active are often unclear. Although the recent development of single-cell RNA sequencing (scRNA-seq) technology has allowed the assessment of the cell type-specific transcriptome, gene regulation that underlies the functional properties of complex diseases is not well understood. Therefore, we performed a comprehensive analysis of cell type-specific gene regulatory mechanisms by integrating publicly available data on cell type-specific epigenome, single-cell transcriptome, and 3D chromatin interactome surrounding human lung cancer. We explored the dynamics of epigenomic profiles of eight major cell types that exist in the tumor environment and the relationships of cell type‒specific regulatory elements with genetic risk variants. By integrating high-resolution chromatin contact maps and scRNA-seq data, we characterize cell type dependent expression profile of putative interaction target genes of lung cancer‒associated variant-harboring *c*REs, and identify a list of potential candidate genes associated with lung carcinoma and their relevant cell type.

## Methods

### Epigenome profiling of regulatory genome elements

Nineteen H3K27ac chromatin immunoprecipitation followed by high-throughput DNA sequencing (ChIP-seq) datasets were downloaded from the Encyclopedia of DNA Elements (ENCODE) database, representing seven cell populations: epithelial (2 primary epithelial cells from mammary gland, 1 primary epithelial cell from prostate), fibroblast (2 primary fibroblast cells from lung and IMR90 cell line), endothelial (2 primary cells from umbilical vein, 1 primary cell from brain microvasculature), T lymphocyte (1 primary T cell, 1 naïve thymus-derived CD4-positive primary cell, and 1 CD8-positive primary cell), natural killer (NK) cell (1 natural killer primary cell), B lymphocyte (2 primary B cells, and GM12989 cell line), myeloid (3 CD14-positive primary monocyte cells) [2,3]. H3K27ac ChIP-seq data for 3 lung cancer cell lines (A549, A427, and H322) were downloaded from the DBTSS database [15]. H3K27ac ChIP-seq data for two alveolar lung epithelial cells representing squamous type 1 (AT1) cells and cuboidal type 2 (AT2) cells were downloaded from NCBI Gene Expression Omnibus (GEO) database (accession code: GSE84273) [16]. Detailed sample information, biosample ID, and library ID for raw ChIP-seq data are described in Supplementary Table 1. The ChIP-seq reads were aligned against the human genome (hg19) using BWA-mem with default parameters. Non-uniquely mapped, low-quality (MAPQ < 10), and PCR duplicate reads were removed. Peak calling of individual ChIP-seq experiments was performed with MACS2 callpeak with a p-value threshold of 1e-5 and with a respective input control used as the background [17]. *c*REs were obtained by selecting H3K27ac peaks located distal to promoters (>2.5 kb from transcription start site [TSS]), merging the peaks across the samples, and stitching peaks within a 3 kb distance. For the quantification of *c*RE activity, reads per million (RPM) values were calculated and quantile normalized across the samples for comparative analysis.

### Collection of genome-wide association study‒single nucleotide polymorphisms associated with lung cancer

A total of 286 genome-wide association study (GWAS)‒single nucleotide polymorphism (SNPs) related to lung cancer were obtained from the NHGRI-EBI GWAS catalog (downloaded on August 20, 2019) [18], targeting nine traits as follows: familial lung adenocarcinoma, familial lung cancer, familial squamous cell lung carcinoma, non‒small cell lung cancer, non‒small cell lung cancer (recurrence rate), non‒small cell lung cancer (survival), small cell lung cancer (survival), small cell lung carcinoma, and squamous cell lung carcinoma. We expanded these tag SNPs by using linkage disequilibrium (LD) information (r^2^ > 0.8). The LD scores were calculated using PLINK for five ethnic populations obtained from 1000 Genomes Phase 3 data. Tight LD associations (r^2^ > 0.8) recurrent in at least three ethnic groups were used for LD expansion. The number of total LD-expanded SNPs was 2,128, and these SNPs were stored in a manner that each of them was traceable back to its parental tag SNP.

### Mapping 3D long-range chromatin interactions

To obtain high-resolution chromatin contact maps in lung tissue, we downloaded *in situ* Hi-C data for A549 (lung carcinoma cell line), IMR90 (lung fibroblast cell line), GM12878 (lymphoblastoid cell line), and HMEC (human mammary epithelial cell line) cells from the ENCODE database [2,3]. Detailed sample information, biosample ID, and library ID for the raw *in situ* HiC data are described in Supplementary Table 2. Raw Hi-C sequenced reads were mapped to the human reference genome (hg19) using BWA-mem (-M option). An in-house script was used to remove low-quality reads (MAPQ < 10), the reads that span ligation sites, chimeric reads, and self-interacting reads in which two fragments are located within 15 kb. The read pairs were merged together as paired-end aligned BAM files, and PCR duplicates were removed with Picard. Statistically significant contacts in Hi-C data were identified at a 5 kb resolution using Fit-Hi-C [19]. We used the default Fit-Hi-C code to calculate the Q-value for each bin pair within a 1 Mb genomic window. A Q-value threshold (Q < 0.01) was used to define significant chromatin contacts.

### Identification of cell type-specific gene expression using scRNA-seq data

The raw UMI count matrix of scRNA-seq data for lung adenocarcinoma was downloaded from NCBI GEO with accession code GSE131907 [20]. Data covering 11 tumors and 11 distant normal lungs were selected and processed by using the Seurat R package v3.2.2 [21]. We discarded cells that expressed < 200 genes. To exclude low-quality cells from our data, we filtered out the cells that expressed mitochondrial genes in > 20% of their total gene expression. In each cell, the gene expression was normalized on the basis of the total read count and log transformed. To align the cells originating from different samples, 5,000 highly variable genes from each sample were identified by the vst method. We found anchors and aligned the samples based on the top 10 canonical correlation vectors. The aligned samples were scaled, and principal component analysis was conducted. Then, the cells were clustered by unsupervised clustering (0.5 resolution) and visualized by tSNE using the top 40 principal components (PCs). Known marker genes were used to assign each subcluster to a corresponding cell population: *EPCAM* and *KRT19* for epithelial cells, *DCN* and *COL1A1* for fibroblasts, *PECAM1* and *CLDN5* for endothelial cells, *CD3D* and *TRAC* for T lymphocytes, *NKG7* and *GNLY* for NK cells, *CD79A* and *IGHM* for B lymphocytes, *LYZ* and *CD68* for myeloid cells, and *KIT* and *MS4A2* for mast cells.

## Results

### Identification of cell type‒specific *c*REs associated with human lung tissue

**Fig. 1.**
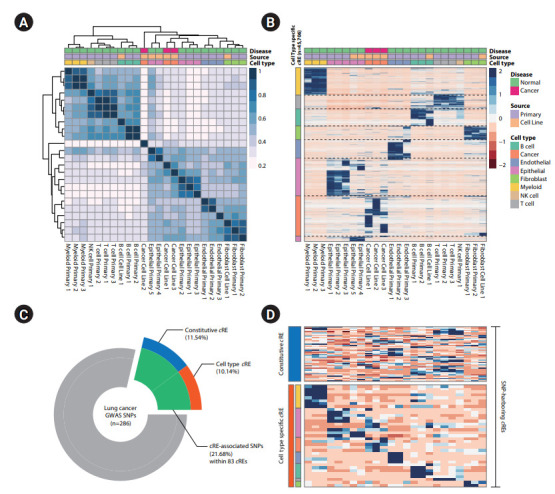
Cell type‒specific association of lung cancer‒related genetic variants with *c*RE. (A) Pearson correlation heatmap illustrating the hierarchical relationship of cell type dependent *c*RE profiles comprising the major cell types in human lung tissue. (B) Heatmap of z-transformed RPM values of cell type‒specific *c*REs. (C) Donut plot illustrating the association of lung cancer GWAS-SNPs with *cis*-regulatory genome elements. (D) Heatmap of z-transformed RPM values of SNP-harboring *c*REs with samples in the column (shown in the same order as the heatmap in Fig. 1B). *c*RE, *cis*-regulatory elements; RPM, reads per million; GWAS, genome-wide association study; SNP, single nucleotide polymorphism; NK, natural killer.

To characterize distal regulatory elements surrounding human lung tissue, we obtained 24 H3K27ac ChIP-seq datasets from the ENCODE, DBTSS, and GEO databases, representing eight major cell types (myeloid cells, T cells, B cells, NK cells, endothelial cells, epithelial cells, lung cancer cells, and fibroblasts) [2,3,15] and identified 86,312 distal *c*REs. The quantification of *c*RE activities in RPM indicated that the samples clustered according to cell type of origin (Fig. 1A), largely into two groups consisting of stromal (epithelial cells, endothelial cells, and fibroblasts) and immune cells (myeloid, NK, T, and B cells). The *c*RE profiles of lung cancer cell lines (A549, A427, and H322) were highly correlated with the epithelial cell type, reminiscent of the cell type of their origin. We identified cell type enriched *c*REs using a quasi-likelihood F test (false discovery rate [FDR] < 0.05) in Bioconductor package EdgeR by contrasting each cell type to the other cell types [22], which resulted in 45,706 cell type‒specific *c*REs (9,707 for lung cancer cells, 14,135 for epithelial cells, 6,952 for myeloid cells, 5,486 for endothelial cells, 4,891 for B cells, 4,447 for fibroblasts, 2,194 for T cells, and 193 for NK cells) (Fig. 1B). This large portion of cell type‒specific *c*REs (52.95%) suggests a dynamic role of regulatory genomic elements in determining cellular identity.

### Characterization of lung cancer‒associated genetic variations with cell type-specific *c*REs

To assess the association of common genetic risk variants for lung cancer with *c*REs with cell type annotation, we collected 286 tag SNPs from the NHGRI-EBI GWAS catalog (downloaded on 2019.08.20) [18] covering nine lung cancer-related traits. Our LD-based association analysis (r^2^ > 0.8) showed that 21.33% (62 of 286) of lung cancer SNPs were linked to *c*REs (Fig. 1C). Among them, 28 tag SNPs were associated with cell type-specific *c*REs (a full list with the corresponding cell type is provided in Table 1), and 34 tag SNPs were linked to constitutive *c*REs (Supplementary Table 3). SNP-harboring *c*REs exhibited cell type‒specific activities, and myeloid (n = 11) and epithelial (n = 10) cell types were recognized by having the most SNP-harboring *c*REs, highlighting a potential role of these cell types in lung carcinogenesis (Fig. 1D).

### Identification of distal target genes of SNP-harboring *c*REs by high-resolution chromatin contact maps

**Fig. 2.**
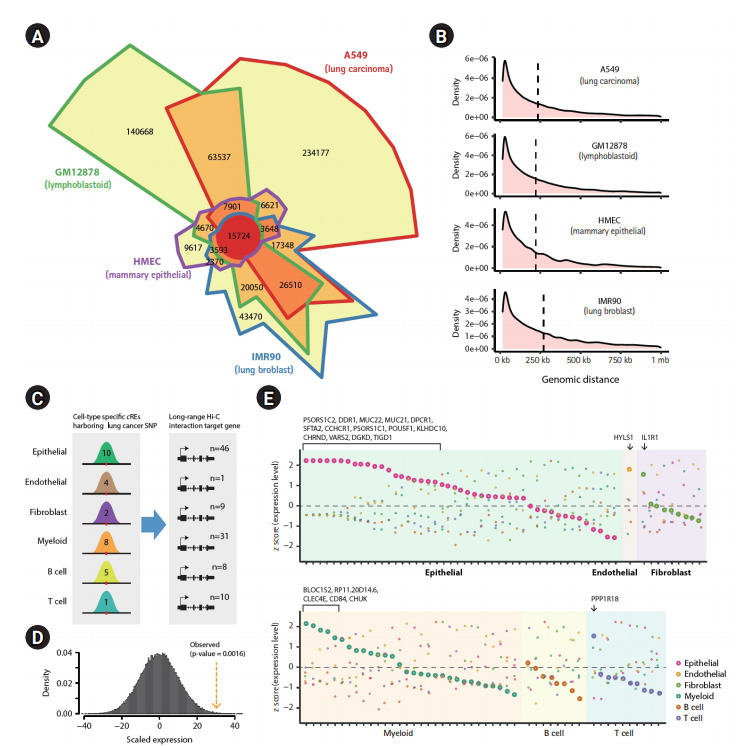
Target gene identification of cell type‒specific *c*REs harboring lung cancer‒associated SNPs based on long-range chromatin interactions. (A) Chow-Ruskey plot with a 5 kb resolution promoter-centered chromatin interactions for GM12878, A549, HMEC, and IMR90 cell lines, representing myeloid, lung cancer, endothelial, and fibroblast cell types, respectively. (B) Density plots illustrating the genomic distance of long-range chromatin interactions obtained from Hi-C data. The dashed line represents the mean distance. (C) A description of the functional link between SNP-harboring *c*REs and inferred target genes through a long-range chromatin contact. (D) Histograms illustrating distribution of the relative expression of randomly selected gene sets based on iterative tests (n = 100,000). Yellow dotted arrows indicate the observed expression of inferred target genes. (E) Gene expression (z-transformed normalized single-cell RNA sequencing counts) of putative target genes of cell type‒specific *c*REs harboring lung cancer-related GWAS-SNPs across the cell types. Genes highlighted in translucent green, brown, purple, orange, yellow, and blue indicates putative targets of cell type‒specific *c*REs in epithelial, endothelial, fibroblast, myeloid, B cells, and T cells, respectively. *c*RE, *cis*-regulatory elements; SNP, single nucleotide polymorphism; GWAS, genome-wide association study.

Despite the enrichment of lung cancer‒associated genetic variants in *c*REs, their biological function in lung cancer is largely unknown due to the lack of information about their functional target genes. We hypothesized that investigating the physical chromatin contacts between promoters and SNP-harboring *c*REs may substantially advance our current knowledge regarding the possible regulatory role of noncoding genetic variants. To this end, we downloaded *in situ* Hi-C data for the A549, IMR90, GM12878, and HMEC cell lines from the ENCODE database [2,3], representing lung cancer, fibroblasts, myeloid cells, and epithelial cells, respectively. We defined long-range chromatin interactions at 5 kb resolution, implementing the Fit-Hi-C algorithm (FDR < 0.01) [19], which resulted in a total of 3,785,594 unbiased, all-to-all chromatin interactions (1,905,639 for A549, 1,207,580 for IMR90, 1,729,755 for GM12878, and 220,529 for HMEC). Focusing on the chromatin interactions connected to the well-annotated protein-coding gene promoters, we identified chromatin interactions anchored within 2.5 kb of a TSS. Comparison of these promoter-centered interactions across the cell types presented a highly dynamic pattern, with lung cancer (A549) and myeloid (GM12878) cells having a large number of unique chromatin interactions, implicating differential spatial arrangements between *c*REs and promoters in those cell types (Fig. 2A). The average distance for long-range chromatin interactions was similar across cell types (234 kb for A549, 222 kb for GM12878, 269 kb for IMR90, and 221 kb for HMEC) (Fig. 2B). Finally, we predicted putative target genes of cell type-specific *c*REs harboring lung cancer‒associated variants by using a union set of chromatin interactions (Fig. 2C). To assess the cell type-specificity of the inferred target genes, we integrated a scRNA-seq dataset generated from lung tissues of 11 healthy individuals and 11 lung cancer patients [20]. We found that the inferred target genes showed a higher gene expression in the corresponding cell type compared to the randomly selected controls (empirical p = 0.0016 from 100,000 iterations) (Fig. 2D). Although the statistical testing for cell type specificity in target gene expression indicated insignificance in enrichment for epithelial and myeloid cell types (Fisher’s exact test; p = 0.1591 for epithelial and p = 0.607 for myeloid), we identified 20 and 5 putative target genes in epithelial and myeloid cells, respectively, with the highest gene expression in the corresponding cell type (Fig. 2E). In addition, we found that *HYLS1*, *IL1R1*, and *PPP1R18* were inferred target genes in endothelial, fibroblast, and T cell, respectively, which also presented a cell type‒specific gene expression. The list of inferred target genes with cell type‒specific gene expression is provided in Table 2. The statistical insignificance in cell type specificity may be the result of the limited number of tested target genes and the insufficient transcripts detected in the corresponding scRNA-seq technique. The improvement of scRNA-seq techniques covering a higher number of transcripts will help make a clear conclusion in this matter. Altogether, the inference of target genes of cell type‒specific, SNP-harboring *c*REs by using 3D chromatin interaction profile provided considerable insights into functionality of lung cancer‒associated GWAS-SNPs.

### Characterization of *DDR1* and *CD84* as potential risk factors associated with lung carcinogenesis

**Fig. 3.**
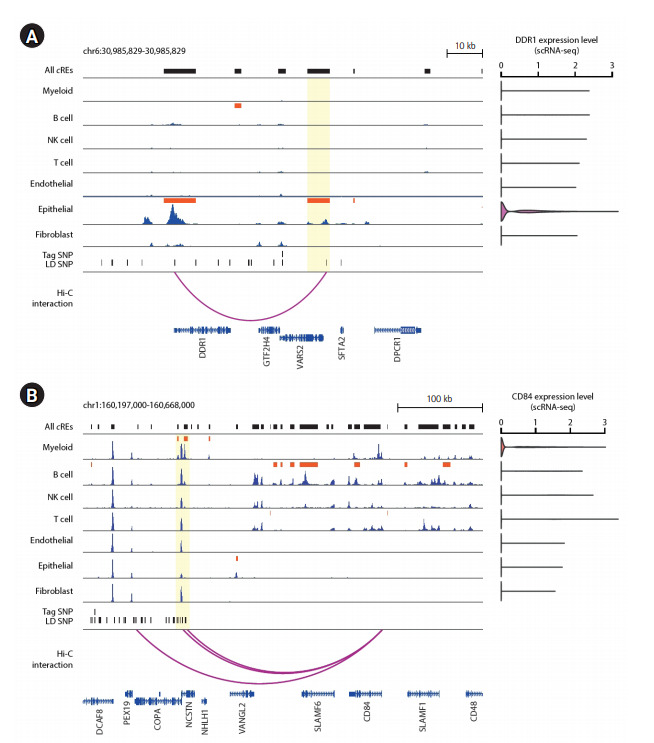
Epigenome landscape of putative target genes of cell type-specific *c*RE harboring lung cancer risk variants. (A) Left: Epigenome browser visualization of the *DDR1* locus (chr6:30,985,829-30,985,829) showing the localization of lung cancer-related GWAS-SNPs, H3K27ac signals over seven individual cell types associated with the human lung, and 5 kb-resolution chromatin loops. The bars in dark orange indicate the location of cell type‒specific *c*REs. The region of epithelial-specific *c*REs sharing lung cancer-related genetic variants is highlighted in translucent yellow. Right: *DDR1* gene expression level across 7 major lung tissue cell types from scRNA-seq data. (B) Left: Epigenome browser visualization of the *CD84* locus (chr1:160,197,000-160,668,000) showing the localization of lung cancer-related SNPs, H3K27ac signals over seven individual cell types associated with the human lung, and 5 kb-resolution chromatin loops. The bars in dark orange indicate the location of cell type dependent *c*REs. The region of myeloid-specific *c*REs sharing lung cancer risk variants is highlighted in translucent yellow. Right: *CD84* gene expression level across 7 major lung tissue cell types from single-cell RNA-seq data. *c*RE, *cis*-regulatory elements; GWAS, genome-wide association study; SNP, single nucleotide polymorphism; scRNA-seq, single-cell RNA-sequencing; LD, linkage disequilibrium.

Since the number of putative target genes with cell type dependent expression was the most prevalent in epithelial and myeloid cells, we sought to pin-point specific risk candidate genes by taking a close examination of the epigenomic landscape surrounding the major cell types in the human lung. First, we found that an epithelial-specific *c*RE (chr6:30,889,573-30,895,783; FDR = 1.61E-05) contains a SNP at chr6:30,894,965, which is linked to a tag SNP at chr6:30,882,415 (rs114274879) based on LD structure. The SNP was significantly associated with squamous cell lung carcinoma (p = 3.0E-16) [23]. The SNP-containing *c*RE was linked to the promoter of *DDR1* by significant long-range chromatin interaction at 50 kb distance, which, in turn, (Fig. 3A). Interestingly, the function of *DDR1* in tumor growth and metastasis displayed an epithelial-specific expression has been previously recognized [28,29]. Next, we identified an additional, myeloid-specific *c*RE (chr1:160,315,750-160,319,868; FDR = 0.030) that contains two LD-expanded SNPs at chr1:160,317,021 and chr1:160,317,619, whose parental tag SNP is located at chr1:160,210,727 (rs2369473). The SNP was reported for its association with squamous cell lung carcinoma (p = 7.0E-06) [23]. The SNP-containing *c*RE is linked to the promoter of *CD84*, a mediator of leukocyte function, by a significant long-range chromatin interaction stretching as far as 230 kb in distance (Fig. 3B). The recently demonstrated function of *CD84* in chronic lymphocytic leukemia cells and their microenvironment may support the potential functional implication of *CD84* in lung carcinogenesis [30]. Furthermore, single-cell transcriptome data indicated that the expression of *CD84* is exclusive in myeloid cells (Fig. 3B). Our results highlight the potential role of *DDR1* and *CD84* in lung carcinogenesis within epithelial and myeloid populations, respectively. The current work involving the functional annotation of lung cancer GWAS-SNPs and the inference of their putative target genes using 3D chromatin contact information effectively expands potential risk candidate genes and their relevant cell types, and offers a rationale for a further investigation of its function in the designated cell type.

## Discussion

We used a comprehensive multi-omics approach that integrates cell type‒specific epigenome, 3D chromatin interactome, and transcriptome data to conduct a functional characterization of lung cancer‒associated GWAS risk variants. It is worth noting that the cellular identity in the global *c*RE landscape is well replicated regardless of tissue origin, genetic background, and culture condition (e.g., primary cells and immortalized cell line), evidenced by the use of publicly available ChIP-seq data representing various cell types in the current study. This led us to effectively find a considerable portion of risk genetic variants associated with *c*REs, taking into consideration the genetic LD. The categorization of lung cancer GWAS-SNPs into corresponding cell types provides additional insights into specific cellular populations responsible for lung carcinogenesis. Our results provide evidence that the identification of the cell type-specific promoter-*c*RE interactome substantially advances the interpretation of GWAS risk variants and broadens the scope for disease risk candidates for lung cancer.

The recent advent of single-nucleus accessible chromatin profiling allows effective identification and characterization of cell populations within human tissues. For example, the single-nucleus ATAC-seq (snATAC-seq) data from the human lung generated by Wang et al. [31] identified six sub-clusters in the epithelial population (AT1/AT2, PNEC, club, basal, and ciliated cells) and cell type‒specific gene regulation associated with viral entry. However, the read-depth and coverage obtained in snATAC-seq data are considerably low for each cell type when compared with bulk ChIP-seq data utilizing primary cells and cell lines. The collection of individual ChIP-seq samples representing major cell types in the human lung, as conducted in this study, may allow a more discrete cell type‒specific investigation of regulatory dynamics. Moreover, the development of scRNA-seq allowed a population-based analysis of transcriptome data. However, it is worth noting that the number of genes detected by scRNA-seq is limited to a few thousand, which largely restricted our scope of investigating the inferred target genes of cell type‒specific *c*REs. Finally, the current work involving the functional annotation of lung cancer GWAS-SNPs and inference of their putative target genes is a notable endeavor, which will provide qualitative insights into disease mechanisms that may be of value in identifying new risk factors developing new approaches for prevention and treatment.

## Figures and Tables

**Table 1.** Annotated list of lung cancer associated GWAS SNPs to cell type–specific *c*REs

Tag SNP	rsID	SNP (P)	Trait	Journal	*c*RE coordinates	Cell type specificity (FDR)	Cell type
chr10.101946033	rs28372851	5.00.E-07	Squamous cell lung carcinoma	Nat Genet (2017) [23]	chr10:101951673‒101953324	1.04.E-02	Myeloid
					chr10:102012793‒102017699	1.20.E-04	Myeloid
					chr10:102049789‒102052812	8.65.E-06	Myeloid
chr10.102048979	rs12765052	1.00.E-06	Squamous cell lung carcinoma	Nat Genet (2017) [23]	chr10:101923384‒101944862	3.86.E-02	Myeloid
					chr10:101951673‒101953324	1.04.E-02	Myeloid
					chr10:102012793‒102017699	1.20.E-04	Myeloid
					chr10:102049789‒102052812	8.65.E-06	Myeloid
chr10.4961021	rs4453114	2.00.E-06	Squamous cell lung carcinoma	Nat Genet (2017) [23]	chr10:5003981‒5007221	1.96.E-02	Epithelial
chr11.125510257	rs113301858	7.00.E-06	Squamous cell lung carcinoma	Nat Genet (2017) [23]	chr11:125543549‒125544766	1.41.E-03	Endothelial
chr1.160210727	rs2369473	7.00.E-06	Squamous cell lung carcinoma	Nat Genet (2017) [23]	chr1:160315750‒160319868	3.07.E-02	Myeloid
chr11.94284529	rs12279741	8.00.E-06	Small cell lung carcinoma	Nat Genet (2017) [23]	chr11:94279690‒94284377	8.37.E-04	Myeloid
chr12.9058562	rs1073160	3.00.E-06	Small cell lung carcinoma	Nat Genet (2017) [23]	chr12:9052330‒9056220	4.30.E-02	Myeloid
chr15.58418128	rs2704201	4.00.E-06	Small cell lung carcinoma	Nat Genet (2017) [23]	chr15:58434184‒58441657	2.98.E-06	Myeloid
chr21.40173528	rs1209950	3.00.E-07	Non‒small cell lung cancer (survival)	J Thorac Oncol (2010) [24]	chr21:40169204‒40174398	4.52.E-02	Cancer
chr2.152481712	rs10174077	1.00.E-06	Squamous cell lung carcinoma	Nat Genet (2017) [23]	chr2:152474585‒152476605	2.78.E-06	Endothelial
chr2.17784157	rs13031455	2.00.E-06	Squamous cell lung carcinoma	Nat Genet (2017) [23]	chr2:17801191‒17803176	1.15.E-06	Epithelial
chr2.225263527	rs6714462	8.00.E-06	Familial squamous cell lung carcinoma	Carcinogenesis (2018) [25]	chr2:225270125‒225272718	1.63.E-02	Endothelial
chr2.233426526	rs1656402	8.00.E-08	Non‒small cell lung cancer (survival)	J Thorac Oncol (2010) [24]	chr2:233453904‒233458464	4.15.E-03	Epithelial
chr2.65832377	rs840781	8.00.E-07	Familial squamous cell lung carcinoma	Carcinogenesis (2018) [25]	chr2:65832165‒65835046	1.09.E-03	B cell
chr3.194858374	rs2131877	2.00.E-08	Non‒small cell lung cancer	Hum Mol Genet (2010)	chr3:194848773‒194855042	7.36.E-03	B cell
chr5.72305846	rs258892	5.00.E-06	Small cell lung carcinoma	Nat Genet (2017) [23]	chr5:72083963‒72085536	1.41.E-03	Epithelial
					chr5:72166471‒72170755	2.59.E-03	Endothelial
chr5.82418056	rs28745309	5.00.E-06	Squamous cell lung carcinoma	Nat Genet (2017) [23]	chr5:82420842‒82421162	4.56.E-02	Cancer
chr6.10415006	rs654351	2.00.E-06	Squamous cell lung carcinoma	Nat Genet (2017) [23]	chr6:10399644‒10415402	1.50.E-02	Epithelial
chr6.26328353	rs34107459	1.00.E-10	Squamous cell lung carcinoma	Nat Genet (2017) [23]	chr6:26327078‒26331665	1.11.E-02	Cancer
chr6.26403036	rs12200782	1.00.E-06	Small cell lung carcinoma	Nat Genet (2017) [23]	chr6:26393324‒26393714	3.84.E-02	Myeloid
chr6.26581258	rs141670911	1.00.E-06	Small cell lung carcinoma	Nat Genet (2017) [23]	chr6:26393324‒26393714	3.84.E-02	Myeloid
					chr6:26462575‒26465582	3.46.E-02	Epithelial
chr6.26651053	rs13201782	2.00.E-08	Squamous cell lung carcinoma	Nat Genet (2017) [23]	chr6:26462575‒26465582	3.46.E-02	Epithelial
chr6.26686131	rs10456332	7.00.E-06	Small cell lung carcinoma	Nat Genet (2017) [23]	chr6:26462575‒26465582	3.46.E-02	Epithelial
					chr6:26757238‒26758412	3.93.E-04	Cancer
					chr6:27144603‒27146930	3.24.E-02	Cancer
chr6.30882415	rs114274879	3.00.E-16	Squamous cell lung carcinoma	Nat Genet (2017) [23]	chr6:30802423‒30803200	9.81.E-04	T cell
					chr6:30848819‒30857882	4.56.E-02	Epithelial
					chr6:30889573‒30895783	2.35.E-03	Epithelial
chr6.32591476	rs112037939	2.00.E-06	Small cell lung carcinoma	Nat Genet (2017) [23]	chr6:32599939‒32607692	6.99.E-04	B cell
chr6.32605884	rs74942078	3.00.E-17	Squamous cell lung carcinoma	Nat Genet (2017) [23]	chr6:32568908‒32579990	2.98.E-02	B cell
					chr6:32599939‒32607692	6.99.E-04	B cell
					chr6:32652218‒32660026	2.53.E-03	B cell
chr6.34923864	rs847845	6.00.E-06	Non-small cell lung cancer	Carcinogenesis (2013) [27]	chr6:34938580‒34938872	5.21.E-03	Fibroblast
chr6.7770511	rs140013431	1.00.E-06	Small cell lung carcinoma	Nat Genet (2017) [23]	chr6:7770218‒7770887	4.90.E-02	Endothelial

GWAS, genome-wide association study; SNP, single nucleotide polymorphism; *c*RE, *cis*-regulatory element; FDR, false discovery rate.

**Table 2.** A list of inferred target genes of SNP-harboring *c*REs with cell-type specific expression

Gene ID	Ensemble ID	*c*RE coordinates	Cell type	Cell type specificity (FDR)	Tag SNP	rsID	SNP (P)	Trait	Journal	Z-transformed gene expression					
										Myeloid	B cell	T cell	Epithelial	Fibroblast	Endothelial
*PSORS1C2*	ENSG00000204538.3	chr6:30848819‒30857882	Epithelial	3.16.E-02	chr6.30882415	rs114274879	3.00E-16	Squamous cell lung carcinoma	Nat Genet (2017) [23]	-0.447	-0.447	-0.447	2.236	-0.447	-0.447
*SFTA2*	ENSG00000196260.3	chr6:30848819‒30857882	Epithelial	3.16.E-02	chr6.30882415	rs114274879	3.00E-16	Squamous cell lung carcinoma	Nat Genet (2017) [23]	-0.412	-0.457	-0.448	2.236	-0.465	-0.454
*DPCR1*	ENSG00000168631.7	chr6:30848819‒30857882	Epithelial	3.16.E-02	chr6.30882415	rs114274879	3.00E-16	Squamous cell lung carcinoma	Nat Genet (2017) [23]	-0.442	-0.451	-0.461	2.236	-0.408	-0.474
*MUC21*	ENSG00000204544.5	chr6:30889573‒30895783	Epithelial	1.61.E-05	chr6.30882415	rs114274879	3.00E-16	Squamous cell lung carcinoma	Nat Genet (2017) [23]	-0.399	-0.469	-0.431	2.235	-0.462	-0.476
*DDR1*	ENSG00000204580.7	chr6:30889573‒30895783	Epithelial	1.61.E-05	chr6.30882415	rs114274879	3.00E-16	Squamous cell lung carcinoma	Nat Genet (2017) [23]	-0.467	-0.456	-0.509	2.232	-0.323	-0.477
*MUC22*	ENSG00000261272.1	chr6:30889573‒30895783	Epithelial	1.61.E-05	chr6.30882415	rs114274879	3.00E-16	Squamous cell lung carcinoma	Nat Genet (2017) [23]	-0.442	-0.497	-0.299	2.231	-0.497	-0.497
*DGKD*	ENSG00000077044.5	chr2:233453904‒233458464	Epithelial	1.07.E-03	chr2.233426526	rs1656402	8.00E-08	Non‒smallcelllungcancer(survival)	J Thorac Oncol (2010) [24]	-0.486	-0.105	-0.336	2.197	-0.681	-0.589
*POU5F1*	ENSG00000204531.11	chr6:30889573‒30895783	Epithelial	1.61.E-05	chr6.30882415	rs114274879	3.00E-16	Squamous cell lung carcinoma	Nat Genet (2017) [23]	-0.696	-0.595	-0.630	2.066	-0.565	0.419
*CCHCR1*	ENSG00000204536.9	chr6:30848819‒30857882	Epithelial	3.16.E-02	chr6.30882415	rs114274879	3.00E-16	Squamous cell lung carcinoma	Nat Genet (2017) [23]	-0.685	-0.730	-0.680	2.062	0.392	-0.358
*PSORS1C1*	ENSG00000204540.6	chr6:30889573‒30895783	Epithelial	1.61.E-05	chr6.30882415	rs114274879	3.00E-16	Squamous cell lung carcinoma	Nat Genet (2017) [23]	-0.737	-0.777	-0.783	2.052	0.090	0.155
*VARS2*	ENSG00000137411.12	chr6:30848819‒30857882	Epithelial	3.16.E-02	chr6.30882415	rs114274879	3.00E-16	Squamous cell lung carcinoma	Nat Genet (2017) [23]	-0.370	-0.835	-1.131	1.951	0.240	0.144
*TIGD1*	ENSG00000221944.3	chr2:233453904‒233458464	Epithelial	1.07.E-03	chr2.233426526	rs1656402	8.00E-08	Non‒small cell lung cancer (survival)	J Thorac Oncol (2010) [24]	-1.161	-0.796	0.255	1.939	-0.406	0.169
*KLHDC10*	ENSG00000128607.9	chr7:130666628‒130685063	Epithelial	2.00.E-02	chr7.130668618	rs6957511	1.00E-06	Squamous cell lung carcinoma	Nat Genet (2017) [23]	-0.355	-1.310	-1.074	1.491	0.584	0.665
*CHRND*	ENSG00000135902.5	chr2:233453904‒233458464	Epithelial	1.07.E-03	chr2.233426526	rs1656402	8.00E-08	Non‒smallcelllungcancer(survival)	J Thorac Oncol (2010) [24]	0.534	-0.964	0.910	1.449	-0.964	-0.964
*C6orf15*	ENSG00000204542.2	chr6:30889573‒30895783	Epithelial	1.61.E-05	chr6.30882415	rs114274879	3.00E-16	Squamous cell lung carcinoma	Nat Genet (2017) [23]	-0.770	-0.770	-0.505	1.368	-0.770	1.352
*PRR3*	ENSG00000204576.7	chr6:30889573‒30895783	Epithelial	1.61.E-05	chr6.30882415	rs114274879	3.00E-16	Squamous cell lung carcinoma	Nat Genet (2017) [23]	-0.377	-1.297	-0.856	1.269	1.131	-0.090
*GIGYF2*	ENSG00000204120.10	chr2:233453904‒233458464	Epithelial	1.07.E-03	chr2.233426526	rs1656402	8.00E-08	Non‒small cell lung cancer (survival)	J Thorac Oncol (2010) [24]	-0.487	-1.368	-1.013	1.242	0.815	0.811
*ZNF322*	ENSG00000181315.6	chr6:27094480‒27096156	Epithelial	2.58.E-02	chr6.26686131	rs10456332	7.00E-06	Small cell lung carcinoma	Nat Genet (2017) [23]	-0.550	-1.106	-1.227	1.209	1.067	0.606
*MDC1*	ENSG00000137337.10	chr6:30889573‒30895783	Epithelial	1.61.E-05	chr6.30882415	rs114274879	3.00E-16	Squamous cell lung carcinoma	Nat Genet (2017) [23]	-0.883	-1.281	-0.782	1.178	0.974	0.794
*HYLS1*	ENSG00000198331.6	chr11:125543549‒125544766	Endothelial	9.66.E-04	chr11.125510257	rs113301858	7.00E-06	Squamous cell lung carcinoma	Nat Genet (2017) [23]	-1.371	-0.807	0.263	-0.207	0.318	1.804
*IL1R1*	ENSG00000115594.7	chr2:102854553‒102858050	Fibroblast	3.09.E-02	chr2.102857739	rs185815317	1.00E-06	Familialsquamouscelllungcarcinoma	Carcinogenesis (2018) [25]	-0.792	-1.055	-1.004	0.611	1.550	0.689
*BLOC1S2*	ENSG00000196072.7	chr10:102012793‒102017699	Myeloid	1.79.E-04	chr10.102048979	rs12765052	1.00E-06	Squamous cell lung carcinoma	Nat Genet (2017) [23]	2.161	-0.253	-0.008	-0.780	-0.438	-0.682
*CLEC4E*	ENSG00000166523.3	chr12:9052330‒9056220	Myeloid	3.18.E-02	chr12.9058562	rs1073160	3.00E-06	Small cell lung carcinoma	Nat Genet (2017) [23]	1.837	-0.689	-0.678	-0.657	-0.695	0.882
*CD84*	ENSG00000066294.10	chr1:160315750‒160319868	Myeloid	2.06.E-02	chr1.160210727	rs2369473	7.00E-06	Squamous cell lung carcinoma	Nat Genet (2017) [23]	1.760	-0.255	0.928	-0.812	-0.814	-0.808
*CHUK*	ENSG00000213341.6	chr10:101951673‒101953324	Myeloid	1.17.E-02	chr10.102048979	rs12765052	1.00E-06	Squamous cell lung carcinoma	Nat Genet (2017) [23]	1.459	-1.436	-0.536	1.058	-0.629	0.084
*PPP1R18*	ENSG00000146112.7	chr6:30802423‒30803200	Tcell	2.70.E-04	chr6.30882415	rs114274879	3.00E-16	Squamous cell lung carcinoma	Nat Genet (2017) [23]	0.965	-0.295	1.539	-1.526	-0.501	-0.183

SNP, single nucleotide polymorphism; *c*RE, *cis*-regulatory element; FDR, false discovery rate.

## Supplementary Materials

Supplementary Table 1. Sample information for ChIP-seq data

Supplementary Table 2. Sample information for *in situ* Hi-C data

Supplementary Table 3. Annotated list of lung cancer-related GWAS-SNPs to constitutive *c*REs
